# *ZAC1* and *SSTR2* Are Downregulated in Non-Functioning Pituitary Adenomas but Not in somatotropinomas

**DOI:** 10.1371/journal.pone.0077406

**Published:** 2013-10-02

**Authors:** Leonardo Vieria Neto, Luiz Eduardo Wildemberg, Leandro Machado Colli, Leandro Kasuki, Nelma Veronica Marques, Aline Barbosa Moraes, Emerson L. Gasparetto, Christina Maeda Takiya, Margaret Castro, Mônica Roberto Gadelha

**Affiliations:** 1 Department of Internal Medicine and Endocrine Unit, Medical School and Clementino Fraga Filho University Hospital, Federal University of Rio de Janeiro, Rio de Janeiro, Brazil; 2 Endocrinology Unit, Federal Hospital of Lagoa, Rio de Janeiro, Brazil; 3 Department of Internal Medicine, Medical School of Ribeirão Preto, Universidade de São Paulo, São Paulo, Brazil; 4 Radiology Unit, Medical School and Clementino Fraga Filho University Hospital, Federal University of Rio de Janeiro, Rio de Janeiro, Brazil; 5 Biofísica Carlos Chagas Filho Institute, Federal University of Rio de Janeiro, Rio de Janeiro, Brazil; Consiglio Nazionale delle Ricerche (CNR), Italy

## Abstract

**Introduction:**

There are few data regarding ZAC1 expression in clinically non-functioning pituitary adenomas (NFPA). Because somatotropinomas and NFPA behave differently with respect to tumor shrinkage during somatostatin analogs (SA) therapy, we sought to compare the ZAC1 and somatostatin receptor (sstr) types 1, 2, 3 and 5 mRNA expression in these two pituitary adenoma subtypes and in normal human pituitaries.

**Methods:**

*ZAC1* and *SSTR* mRNA expression levels were evaluated using real-time RT-PCR (TaqMan) in 20 NFPA and compared with the expression levels in 23 somatotropinomas and five normal pituitaries. The NFPA invasiveness was evaluated using magnetic resonance imaging with Hardy’s modified criteria. Ki-67 and p53 were evaluated using immunohistochemistry.

**Results:**

A total of 20 patients with NFPA [6 males, median age 56 years (range: 30-78)], 23 with acromegaly [12 males, median age 43 years (range: 24–57)] and five normal pituitaries [4 males, median age 48 years (range: 36–54)] were included. Four of the patients (20%) had Hardy’s grade 2 tumors; all of the others had Hardy’s grade 3 tumors. The Ki-67 median expression was 2.35 (range: 0.2–9.23), and only four of the tumors (20%) were positive for p53. The *ZAC1* mRNA expression was significantly lower in NFPA than in somatotropinomas and in normal pituitaries (p<0.001 for both), as well as the *SSTR2* (p=0.001 and 0.01, respectively). The *SSTR3* expression was higher in the NFPA than in the somatotropinomas and in the normal pituitaries (p=0.03 and 0.02, respectively). No correlation was found between the *ZAC1* mRNA expression and the tumor invasiveness, Ki-67 and p53.

**Conclusion:**

ZAC1 and SSTR2 are underexpressed and SSTR3 is overexpressed in NFPA compared to those in somatotropinomas and in normal pituitaries, which might explain the lack of tumor shrinkage that is observed in response to commercially available SA therapy in patients with NFPA.

## Introduction

Somatostatin receptors (SSTR) are G-protein-coupled receptors that are encoded by five separate genes (*SSTR1*–*5*) [1]. All of the five genes are expressed in normal adult human pituitaries, although *SSTR4* mRNA has only been detected at extremely low levels [1]. In pituitary adenomas, SSTR expression has been reported to be highly variable within and between tumor subtypes [2,3]. In clinically non-functioning pituitary adenomas (NFPA), the *SSTR2* and *3* mRNA transcripts were expressed in the majority of tumors, whereas the *SSTR1,* *4* and *5* mRNA transcripts were expressed in a subset of the tumors. The *SSTR3* transcript showed the highest expression level, followed by the *SSTR2* transcript; the *SSTR1*, *4* and *5* transcripts exhibited low levels of expression [2]. In somatotropinomas and normal pituitaries, the *SSTR2* and *5* mRNA transcripts and proteins were expressed in all of the samples [2–6]. The *SSTR5* mRNA transcript exhibited the higher level of expression, followed by *SSTR2, 3,* *1* and *4* [2].

The somatostatin analogs octreotide and lanreotide bind preferentially to SSTR2 (but also to SSTR3 and SSTR5) and are the mainstay in the treatment of acromegaly [7]; however, they have shown a lack of efficacy in NFPA [8]. Studies in GH3 cells demonstrated that the antiproliferative action of octreotide is mediated by an induction of *Zac1* (zinc finger protein which regulates apoptosis and cell cycle arrest) expression [9–12]. ZAC1 is a seven zinc finger protein that acts as both a transcription factor and a coregulator [13]. Its gene is located in the chromosome 6q24-25, which is a frequent site of loss of heterozygosity (LOH) in several tumors, such as pituitary adenomas [14], ovarian cancer [15], breast cancer [16], pheochromocytoma [17] and hemangioblastoma [18]. It is highly expressed in normal human pituitaries but shows reduced expression in pituitary adenomas, especially NFPA [14,19]. In this pituitary adenoma subtype, ZAC1 immunoexpression has been found to be inversely correlated with tumor recurrence after surgery [20].

To better understand the lack of efficacy of the SSTR2 somatostatin analogs in NFPA, we evaluated the mRNA expression levels of *ZAC1* and SSTR subtypes 1, 2, 3 and 5 in NFPA and compared them to those of somatotropinomas and normal pituitaries. In addition, we correlated these expression levels with those of the proliferative markers Ki-67 and p53 and with the invasiveness of the tumors.

## Patients and Methods

This study was approved by the ethics committees of the Clementino Fraga Filho University Hospital/Medical School – Federal University of Rio de Janeiro and of the Ribeirao Preto Medical School - University of São Paulo. This study has been conducted according to the principles expressed in the Declaration of Helsinki. All of the patients signed informed consent forms.

### Patients and tumors

Tumor samples from drug-naïve unselected patients with NFPA were included. The diagnosis of NFPA was based on the presence of a magnetic resonance image (MRI) of the sella turcica with an adenoma visualized in the absence of symptoms suggesting hormone hypersecretion, in addition to biochemical confirmation of a normal or hypofunctioning pituitary. This diagnosis was confirmed by a histopathological examination, and immunohistochemistry revealed either negative immunostaining for all of the anterior pituitary hormones or positive immunostaining for LH, FSH and/or the alpha-subunit. We also studied tumor samples from drug-naïve acromegalic patients and normal human pituitaries for comparison purposes. The diagnosis of acromegaly was based on the presence of classic clinical features associated with a lack of GH suppression to <1.0 µg/L during an oral glucose tolerance test and a high age-matched IGF-I level. A positive GH staining of the tumor specimen upon immunohistochemical examination was required to confirm the etiology. Five normal human pituitary glands were obtained during autopsy following accidental deaths, with no clinical or pathological evidence of endocrine disorders. The time between death and sample collection was 6–12 h. Pathological examination excluded the presence of pituitary adenoma/hyperplasia or metastasis.

The NFPA were classified as invasive or non-invasive according to radiological criteria using Hardy’s modified criteria [21] [19]. All of the tumors were evaluated using preoperative MRI, and all of the images were analyzed by the same radiologist (ELG), who was blinded to the RT-PCR and immunostaining results. Briefly, grade 1 tumors are microadenomas (<1 cm diameter), and grade 2 tumors are macroadenomas (>1 cm diameter) with or without suprasellar extension. Grade 3 tumors are locally invasive, with evidence of bone destruction and the presence of the tumor within the sphenoid and/or cavernous sinus. Grade 4 tumors exhibit central nervous system/extracranial spread with or without distant metastases. Grade 3 and 4 tumors are considered invasive.

### Assessment of ZAC1 and SSTR1, 2, 3 and 5 mRNA expression levels using real-time RT-PCR

The total RNA was extracted from a 30-mg fresh tissue sample obtained during transsphenoidal surgery using the RNeasy® Mini Kit (Qiagen, Valencia, CA). To avoid contamination by genomic DNA, the RNA samples were treated with an RNase-free DNase Set (Qiagen), as specified in the manufacturer’s protocol. The integrity of the total RNA was confirmed using standard agarose gel electrophoresis with ethidium bromide staining. The RNA quantification was performed using a spectrophotometer NanoDrop 1000 (NanoDrop Technologies, Wilmington, Delaware, USA) at a wavelength of 260 nm. The ratio of the absorbances at 260 nm and 280 nm was used to verify the purity of the RNA. The synthesis of first-strand cDNA suitable for PCR amplification was performed using the enzyme MultiScrib® kit and high capacity cDNA Reverse Transcription (Applied Biosystems, CA, USA) with 500 ng of RNA (final volume 20 μL), according to protocols supplied by the manufacturer.

To control for variations in the amount of sample and the RNA quality used in the RT reaction and the efficiency of the RT reaction, the expression levels of three housekeeping genes [*TATA-binding protein (TBP*)*, β-glucuronidase (GUS*)* and phosphoglycerate kinase-1 (PGK1*)] were determined for each sample. To determine whether these genes were appropriate to be used as internal controls, the stability of expression was calculated using the GeNorm 3.3 visual basic application for Microsoft Excel, as previously developed and validated by Vandesompele et al. [22]. This program calculates the average pairwise variation of a particular gene with all of the other control genes, allowing the elimination of the worst-scoring control genes and recalculation. The geometric means of the copy numbers for the most stable genes are then used as a normalization factor. In the current study, the three housekeeping genes were confirmed as being stable.

The real-time RT-PCR reactions were performed using the TaqMan® method. The amplification reactions were made in duplicate, using 96-well plates with 0.5 µL of primers + probe, 5 µL of master mix, 2.5 µL of cDNA and 2.0 µL of DEPC water. The thermal cycling profile consisted of an initial temperature of 50°C for 2 minutes, followed by a denaturation at 95°C for 10 minutes and then 40 successive cycles at 95°C for 15 seconds and 60°C for 1 minute. The TaqMan® assays used in each reaction are identified in Table 1. The total RNA sample that was not reverse-transcribed was run to control for genomic and/or technical contamination. To determine the inter-assay variation, one identical sample was used in all of the RT-PCR reactions. The intra-assay variation was controlled for by performing the reaction in duplicate for each sample. A difference of 1.5 standard deviations was considered to be an acceptable intra-assay variation.

**Table 1:** Quantitative real-time RT-PCR assays.

**Gene**	**TaqMan assay**
*ZAC1*	Hs00414677_m1
*SSTR1*	Hs00265617_s1
*SSTR2*	Hs00990356_m1
*SSTR3*	Hs01066399_m1
*SSTR5*	Hs00990408_s1
*TBP*	Hs_00427621_m1
*GUS*	Hs_00939627_m1
*PGK1*	Hs_99999906_m1

The amplification curves were visualized using the software ABI PRISM® 7500, and the detection threshold was set in the exponential phase of the curve. The point at which each amplification curve crosses the threshold serves as a basis for comparison between the samples, using the same threshold for all of the reactions (cycle threshold, Ct). The expression analysis was performed using QPCR software [23], and the efficiency for each reaction was calculated using the linear regression model with the software LingRegPCR [24].

After establishing the Ct for each sample, the results were normalized by subtracting the Ct for the target gene (*ZAC1* and *SSTR* subtypes) from the Ct for endogenous control [the geometric mean of the Ct values for each sample of each gene was calculated to obtain a value used for normalization (normalization factor)] of the results, generating the ΔCt (Ct_sample (target gene)_ – Ct_sample (housekeeping genes)_). The normalized results (ΔCt) were then submitted to a calibration process. For that process, we used the median ΔCt of the five normal pituitary samples, obtaining the ΔΔCt (ΔCt_sample_ - ΔCt_calibrator_). Consequently, the relative expression of each gene in the sample was given by the formula (1 + efficiency)^-ΔΔCt^. The median values of (1 + efficiency)^-ΔΔCt^ obtained from the tumor samples (NFPA and somatotropinoma) were compared to the median value of (1 + efficiency)^-ΔΔCt^ from the normal pituitary tissue samples, obtaining a value that shows how many times (fold change) a gene is over- or underexpressed in the tumor tissue compared to normal pituitary tissue (relative expression).

To exclude the possibility of contamination of NFPA and somatotropinomas with normal pituitary tissue, the expression levels of the transcription factors SF1, Tpit and Pit1 were evaluated using conventional RT-PCR. In humans, Pit-1 expression is restricted to somatotrophs, mammosomatotrophs, lactotrophs, and thyrotrophs. The transcription factor T-pit is expressed selectively in proopiomelanocortin (POMC)-expressing corticotroph cells of the pituitary. Thus, in NFPA samples, we used these transcription factors to evaluate contamination with somatotrophs, mammosomatotrophs, lactotrophs, thyrotrophs, and corticotrophs. SF-1 expression in the pituitary was demonstrated to be restricted in gonadotroph lineage. So, in somatotropinoma samples, we used SF-1 and T-pit to assess contamination with gonadotrophs and corticotrophs.

### Immunohistochemistry

Immunohistochemistry for Ki-67 and p53 was performed according to the protocol previously described [25]. Briefly, the tissues were submitted to heat-mediated antigen retrieval in Dako® Retrieval buffer (Dako, Carpinteria, CA, USA), pH 9.0 in a steamer for 30 minutes for ki-67 and in 0.01 M citric acid buffer, pH 6.0 in a microwave for 10 minutes at 750 W for p53. The primary antibody incubation was performed overnight with antibodies directed against the Ki-67 antigen (1:100, Dako, MIB-1 clone, cat. number M-7240) and p53 (1:100, Santa Cruz Biotechnologies, Santa Cruz, CA, USA, DO1 clone, cat. number sc-126), which were detected using the EnVision peroxidase kit (Dako, K1491), followed by the chromogen substrate diaminobenzidine (Liquid DAB, Dako K3468). Quantification was performed by counting the immunolabeled and unlabeled nuclei and then calculating the percentage of positive cells (Labeling Index – LI).

### Statistical analysis

Analyses were performed using SPSS version 16.0 for Windows (SPSS Inc., Chicago, IL). In the descriptive analysis, the categorical variables were expressed by their percentages and frequencies, whereas the numerical variables were expressed by their medians (minimum and maximum). The Kruskal-Wallis test was used to compare the numerical variables among three groups and the Mann–Whitney test was performed for comparison between two groups. The correlations between numeric variables were studied using the Spearman’s correlation test. A p-value of less than 0.05 was considered significant.

## Results

### Patients and samples characteristics

Twenty patients with NFPA [6 males, median age 56 years old (30-78)], 23 with acromegaly [12 males, median age 43 years old (24-57)] and five normal pituitaries [4 males, median age 48 years old (36-54)] were included in the study. All of the patients with NFPA presented macroadenomas; two (10%) had null cell adenomas, and the others showed immunopositivity for LH, FSH and/or α-subunit (gonadotropinomas). Four of the patients (20%) had Hardy’s grade 2 tumors, and all of the others had Hardy’s grade 3 tumors. All of the tumors exhibited immunostaining for Ki-67, with a variable level of expression [median 2.35 (0.2-9.23)], whereas only four (20%) of the tumors were positive for p53 (Table 2).

**Table 2:** Demographic, ZAC1, somatostatin receptors subtypes and Ki-67 data in non-functioning pituitary adenomas.

	**Age**	**Sex**	**IHC**	**Hardy**	***ZAC1***	***SSTR1***	***SSTR2***	***SSTR3***	***SSTR5***	**Ki-67**	**p53**
**Non-invasive**											
1	54	F	G	2	0.004	0.016	6.924	0.009	0.010	0.20	0
2	74	F	G	2	0.002	7.103	0.102	15.084	4.387	1.16	0
3	30	F	G	2	0.039	43.72	0.066	3.540	34.534	5.66	0.26
4	50	F	G	2	0.028	0.038	0.231	2.312	0.055	2.71	0
Median	52				0.016	3.571	0.166	2.926	2.221	1.935	0
**Invasive**											
5	39	F	G	3	0.001	0.010	0.052	0.726	0.042	9.23	0.63
6	58	F	G	3	0.018	10.193	2.589	2.421	2.988	1.99	0
7	57	F	G	3	0.027	4.741	1.314	11.639	5.256	2.36	0
8	58	M	G	3	0.001	0.200	0.08	5.304	0.298	1.75	0
9	52	M	G	3	0.003	0.227	0.041	7.189	0.106	3.00	0
10	56	F	G	3	0.010	0.968	0.017	3.154	0.663	3.02	0
11	44	F	G	3	0	3.477	0.039	7.372	2.472	2.89	0
12	68	M	G	3	0.068	1.663	0.088	1.195	1.378	2.32	0
13	66	F	N	3	1.267	0.026	0.014	0.018	0.093	4.07	0
14	56	F	G	3	0.002	0.174	0.065	4.981	0.275	2.62	0
15	64	M	G	3	0.038	0.247	0.151	13.486	0.323	1.85	1.1
16	34	F	G	3	0.001	0.245	0.013	9.105	0.328	2.33	0
17	75	M	G	3	0.015	1.059	0.27	5.677	0.964	2.00	0.7
18	50	M	N	3	0.024	0.048	0.257	4.166	2.710	1.15	0
19	54	F	G	3	0.005	0.299	0.024	2.795	0.470	2.20	0
20	78	F	G	3	0.001	9.538	0.165	4.028	7.974	0.28	0
Median	56.5				0.008	0.273	0.073	4.574	0.567	2.325	0

Legend: F = female; M = male; IHC = immunohistochemistry for LH, FSH and α-subunit; G = gonadotropinoma; N = null cell adenoma; *SSTR* = somatostatin receptor

In the acromegalic patients, two (8.7%) harbored a microadenoma. The median GH at diagnosis was 8.1 ng/mL (0.9-235), whereas the median IGF-I was 3.1 times the upper limit of the normal range (1.3-6.9).

### ZAC1 mRNA expression

**Figure 1:**
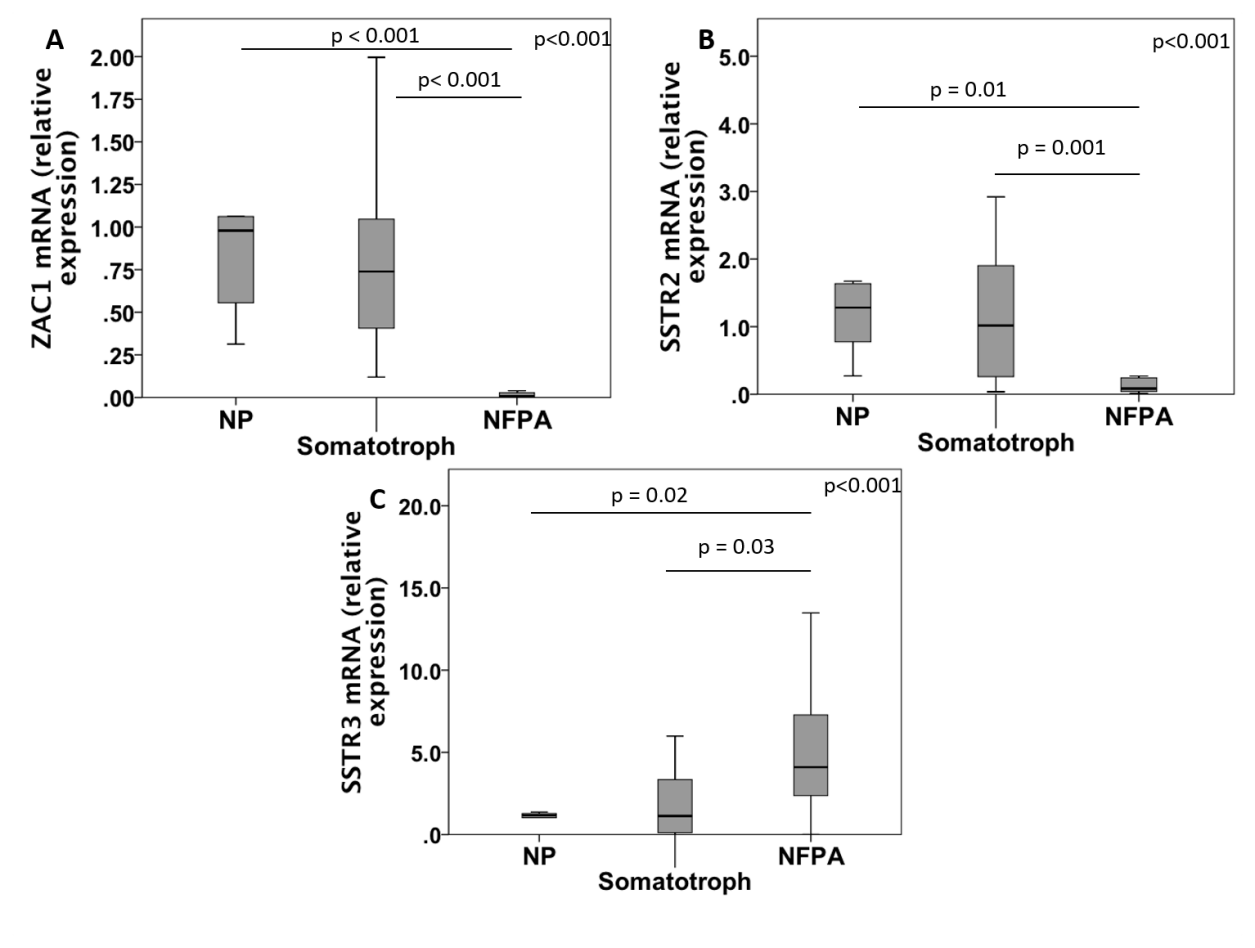
ZAC1, SSTR2 and SSTR3 expression in NFPA, somatotropinomas and normal pituitaries. Comparison of *ZAC1* (A), *SSTR2* (B) and *SSTR3* (C) mRNA expression levels among normal pituitaries (NP), somatotropinomas (somatotroph) and non-functioning pituitary adenomas (NFPA). The outliers were excluded. The Kruskall-Wallis test was used to compare the mRNA expression among the three groups and the Mann-Whitney test for comparison between NFPA and normal pituitaries and somatotropinomas.

The *ZAC1* mRNA was expressed at very low levels in all but one of the NFPA, with a median of 0.007, varying from 0 to 1.267 (Table 2). In the somatotropinomas, this expression was variable; the median was 0.739 and it varied from 0.12 to 3.321 (Table 3). The ZAC1 mRNA expression was different among the three groups (p < 0.001). The expression level was 106 times lower than in the somatotropinomas and 140 times lower than in the normal pituitaries (p values < 0.001 for both) [Table 4; Figure 1].

**Table 3:** Demographic, ZAC1 and somatostatin receptors subtypes data in somatotropinomas.

	**Age**	**Sex**	***ZAC1***	***SSTR1***	***SSTR2***	***SSTR3***	***SSTR5***
1	30	F	1,151	0,007	0,374	0,005	1,802
2	37	F	0,785	0,033	15,505	0,11	0,005
3	36	M	0,307	0,038	0,122	324,759	0,722
4	32	M	0,384	3,694	0,234	0,126	0,014
5	56	F	0,352	0,026	0,535	0,045	0,471
6	53	F	3,321	0,141	0,264	8,315	1,201
7	31	F	0,959	0,036	0,256	10,028	0,66
8	42	M	0,302	0,018	2,869	0,454	0,012
9	57	M	0,739	0,09	0,321	12,857	4,046
10	50	F	1,605	0,034	1,026	4,101	0,337
11	24	M	0,429	0,008	6,509	0,002	0,03
12	42	F	0,624	0,092	1,586	2,094	0,943
13	43	F	1,041	0,068	1,793	5,988	0,648
14	37	F	0,519	0,232	0,04	0,988	0,526
15	43	M	1,996	0,097	2,919	1,131	1,261
16	36	M	0,814	1,012	0,319	1,629	3,362
17	40	F	0,626	7,463	0,037	2,582	2,53
18	55	M	0,701	1,502	0,199	0,159	2,233
19	50	M	0,12	5,146	1,727	1,444	0,534
20	48	M	0,936	4,144	1,855	0,055	4,356
21	49	F	1,052	5,898	1,017	0,169	3,012
22	43	M	1,466	1,227	1,951	2,308	0,44
23	46	M	0,318	0,077	22,949	0,113	0,346
Median	43		0,739	0,092	1,017	1,131	0,66

Legend: F = female; M = male; SSTR = somatostatin receptor

**Table 4:** ZAC1 and somatostatin receptor subtype mRNA expression levels in non-functioning pituitary adenomas, somatotropinomas and normal pituitaries.

	**NFPA**	**Somatotropinomas**	**Normal pituitaries**	**p value†**
*ZAC1*	0.007 (0-1.267)	0.739 (0.12-3.321)^a^	0.979 (0.313-2.089)^a^	< 0.001
*SSTR1*	0.273 (0.01-43.72)	0.092 (0.007-7.463)	0.903 (0.299-1.719)	NS
*SSTR2*	0.084 (0.013-6.924)	1.017 (0.037-22.949)^b^	1.281 (0.272-1.672)^c^	< 0.001
*SSTR3*	4.097 (0.009-15.084)	1.131 (0.002-324.759)^d^	1.171 (0.525-1.366)^e^	< 0.001
*SSTR5*	0.566 (0.01-34.534)	0.66 (0.005-4.356)	0.858 (0.487-2.033)	NS

Data expressed in median (min-max).

The Kruskall-Wallis test (†) was used to compare the mRNA expression among the three groups.

The Mann-Whitney test was used for comparison between NFPA and normal pituitaries and somatotropinomas (a = <0.001; b = 0.001; c = 0.01; d = 0.03; e = 0.02)

### *SSTR* subtype mRNA expression levels

The four SSTR subtypes were expressed in all of the NFPA and somatotropinomas with a variable level of expression (Tables 2 and 3). The *SSTR3* was higher in the NFPA than in the somatotropinomas and the normal pituitaries (p values of 0.03 and 0.02, respectively). On the other hand, *SSTR2* was lower in the NFPA than in the somatotropinomas and normal pituitaries (p values of 0.001 and 0.01, respectively) [Table 3; Figure 1]. No correlation was found between the *ZAC1* and the *SSTR2* or the other *SSTR* subtype mRNA expression levels in the NFPA.

### Comparison of ZAC1 expression with Ki-67, p53 and invasiveness

No correlation was found between *ZAC1* and Ki-67 or p53. In addition, the *ZAC1* expression level was similar in the invasive and non-invasive NFPA.

## Discussion

In this study, we demonstrated for the first time that *ZAC1* mRNA was underexpressed in NFPA relative to normal human pituitaries and somatotropinomas using a quantitative technique (real-time RT-PCR). Similar data have been previously reported; however, that studies involved a limited number of patients and a semi-quantitative technique (conventional RT-PCR) [14,19] [12,17]. On the other hand, ZAC1 mRNA expression was similar in somatotropinomas and normal human pituitaries. In respect to the SSTR, lower *SSTR2* and higher *SSTR3* mRNA expression levels were found in the NFPA compared with both the normal pituitaries and the somatotropinomas. A previous study from our group also found a lower SSTR2 mRNA expression in NFPA in comparison to somatotropinomas, however, the SSTR3 was similar in these adenoma subtypes [2].

Tumor shrinkage has been observed in approximately 40% of somatotropinomas and in 11–13% of NFPA [8] after treatment with octreotide and lanreotide [26], which bind preferentially to SSTR2. This effect occurs despite the fact that the SSTR subtype with the highest level of expression in these tumors is SSTR3, which is the main SSTR subtype associated with apoptosis [27]. Theodoropoulou et al. [10] demonstrated that, in GH3 cells (a rat somatomammotroph pituitary cell line that expresses SSTR1 and 2), the anti-proliferative action of octreotide occurred by increasing *Zac1* gene expression. In addition, *Zac1* gene knockdown abolishes the anti-proliferative effect of octreotide. These data indicate that ZAC1 is involved in the anti-proliferative action of somatostatin analogs and that its expression is necessary for this effect. Consequently, the low ZAC1 expression in NFPA may explain, at least in part, the lack of response of these tumors to somatostatin analog treatment. However, apart from the SSTR or ZAC1 expressions, differences in the pathogenesis of somatotropinomas and NFPA are also likely to account for the different response to somatostatin analogs in these adenoma subtypes.

On the other hand, Theodoropoulou et al. [28] recently also demonstrated in GH3 cells that *Zac1* inhibition by siRNA suppressed *Sstr2* expression. In contrast, increasing *Zac1* expression by treatment with the PI3K inhibitors LY294002 and wortmannin led to an increase in *Sstr2* expression. Although these data was evaluated in GH3 cells, which is not an appropriate model for NFPA, we hypothesize that, analogously, the low ZAC1 expression might contribute to explain the low SSTR2 expression observed in NFPA. However, we did not find correlation between *ZAC1* and *SSTR2* expression in NFPA or somatotropinomas.

The transfection of *Zac1* in LLC-PK1 (porcine kidney epithelial) and SaOs-2 (human osteogenic sarcoma) cell lines caused a substantial reduction in cell proliferation, comparable with transfection with p53 [26]. Whereas the ablation of *Zac1* gene expression using antisense treatment in AtT-20 (mouse corticotroph pituitary tumor) and TtT/GF (folliculo-stellate) cell lines stimulated DNA synthesis in 36–50% [29]. These data may indicate a possible theoretical role of the low ZAC1 expression in the pathogenesis of NFPA, however, this hypothesis still needs to be further studied.

We examined the proliferative markers Ki-67 and p53 using immunohistochemistry, and no correlations were observed of these markers to the *ZAC1* expression levels in NFPA. This result may be explained by the very low *ZAC1* expression in all of the tumors except one, suggesting that low expression of *ZAC1* is a characteristic of NFPA as a group. Curiously, the tumor with the highest *ZAC1* expression also presented a high Ki-67 LI.

Noh et al. [20] analyzed several markers associated with cell proliferation or apoptosis in 35 NFPA and tumor recurrence. They found that recurrent NFPA had higher levels of Ki-67 and TUNEL labeling indexes. In addition, high level of expression of phospho-Akt, phospho-p44/42 MAPK and PTTG1 were associated with early recurrence. On the other hand, phospho-CREB and ZAC1 expression levels were inversely associated with recurrence. We compared the *ZAC1* expression levels with the tumor invasiveness, but no association was found. A previous study from our group already compared the levels of Ki-67 and p53 between invasive and non-invasive NFPA in a larger subset of tumors, which included some of the tumors described here (16 ACNF and 5 somatotropinomas), and found similar results [25]. This lack of an association between the *ZAC1* levels and invasiveness may be due to the very low *ZAC1* mRNA expression, as was the case for Ki-67 and p53. Another explanation is that the high frequency of invasive tumors in our series may have led to a type II error. A possible cause for this high frequency is that this series examined tumors that required operations. Smaller and non-invasive tumors frequently do not require operations, so in a series of operated tumors, the incidence of invasive tumors would be expected to increase. Another explanation may be that the study was held at a referral center, which receives more advanced and complicated cases.

As stated above, the SSTR3 is the main subtype associated with apoptosis [27], and the fact that NFPA express this receptor in a large amount may be of interest with the development of new SA, with broader spectrum of binding to SSTR, such as pasireotide, or SSTR3 specific. Florio et al. [30] demonstrated in vivo, using matrigel sponge model, that a SSTR3 specific agonist, L-796778, significantly inhibited angiogenesis. This effect was reversed by a SSTR3 antagonist, BN81658. Data about pasireotide treatment of patients with NFPA is still lacking. In this series, we did not find any difference in gender, age, ZAC1 or Ki-67 between adenomas with high or low SSTR3 expression (data not shown).

In conclusion, we found that *ZAC1* and *SSTR2* expression levels are reduced in NFPA compared to somatotropinomas and normal pituitaries. Despite the function of *ZAC1* as a tumor suppressor gene, we did not find any correlations between its level of expression and those of proliferative markers or invasiveness. However, the low expression levels of *ZAC1* and *SSTR2* may be involved in the resistance to somatostatin analogs that is observed in these tumors, partially justifying the failure of tumor shrinkage that is observed in NFPA compared to somatotropinomas. 
