# Correction: *ZAC1* and *SSTR2* Are Downregulated in Non-Functioning Pituitary Adenomas but Not in somatotropinomas

**DOI:** 10.1371/annotation/c55f4d48-d393-4ff5-b363-c9fc85bfbd93

**Published:** 2013-10-08

**Authors:** Leonardo Vieira Neto, Luiz Eduardo Wildemberg, Leandro Machado Colli, Leandro Kasuki, Nelma Veronica Marques, Aline Barbosa Moraes, Emerson L. Gasparetto, Christina Maeda Takiya, Margaret Castro, Mônica Roberto Gadelha

As a result of issues in the production process, there was an error in the name of the first author.

The correct name of this author is: Leonardo Vieira Neto

The correct citation is: Vieira Neto L, Wildemberg LE, Colli LM, Kasuki L, Marques NV, et al. (2013) *ZAC1* and *SSTR2* Are Downregulated in Non-Functioning Pituitary Adenomas but Not in somatotropinomas. PLoS ONE 8(10): e77406. doi:10.1371/journal.pone.0077406 

